# A Novel Ribozyme-Based Prophylaxis Inhibits Influenza A Virus Replication and Protects from Severe Disease

**DOI:** 10.1371/journal.pone.0027327

**Published:** 2011-11-14

**Authors:** Julie Motard, Ronan Rouxel, Alexandra Paun, Veronika von Messling, Martin Bisaillon, Jean-Pierre Perreault

**Affiliations:** 1 Département de biochimie, RNA group/Groupe ARN, Faculté de médecine et des sciences de la santé, Université de Sherbrooke, Sherbrooke, Canada; 2 INRS-Institut Armand-Frappier, Université du Québec, Laval, Canada; Beckman Research Institute of the City of Hope, United States of America

## Abstract

Influenza A virus seasonal outbreaks and occasional pandemics represent a global health threat. The high genetic instability of this virus permits rapid escape from the host immune system and emergence of resistance to antivirals. There is thus an urgent need to develop novel approaches for efficient treatment of newly emerging strains. Based on a sequence alignment of representatives from every subtype known to infect humans, we identified nucleic acid regions that are conserved amongst these influenza A populations. We then engineered SOFA-HDV-Ribozymes as therapeutic tools recognizing these conserved regions to catalytically cleave the corresponding viral mRNA targets. The most promising ribozymes were chosen based on an initial *in silico* screening, and their efficacy was assessed using *in vitro* cleavage assays. Further characterization of their antiviral effect in cell culture and in mice led to the gradual identification of prophylactic SOFA-HDV-Ribozyme combinations, providing proof-of-principle for the potential of this novel strategy to develop antivirals against genetically highly variable viruses.

## Introduction

Influenza A virus belongs to the *Orthomyxoviridae* family, and its genome consists of eight fragments of single-stranded RNA of negative polarity. The annual seasonal outbreaks as well as occasional pandemics constitute major public health threats. During a typical influenza season, 5–15% of the population in the United States is infected, causing 3 to 5 million people to suffer from severe illness. Major genetic changes in the circulating strain can result in global pandemics, which have happened three times in the 20^th^ century, with the most devastating pandemic killing over 40 million people in 1918–1919 [1]. Influenza pandemics remain a constant threat because of the unpredictability of the timing, the nature, and the virulence of the next major antigenic shift or drift [2]–[4].

Currently, the annual vaccine containing the predicted predominant strains represents the most effective tool for influenza control. Unfortunately, the high genetic variability of the virus renders the protection incomplete. In the case of a newly emerging strain, vaccination is only available a few months after the first appearance, leaving the population vulnerable during the crucial early phase of a pandemic. There are several influenza-specific antiviral drugs available, namely neuraminidase inhibitors and M2-ion channel blockers, which can be associated with important gastrointestinal and sometimes neurological side effects [2], [5]–[7]. Furthermore, drug-resistant strains have emerged and rapidly spread during the last years [8]–[10]. New antiviral strategies based on RNA interference show encouraging effectiveness both in cell cultures and in animal models [11]–[18]. However, problems of potency, specificity, and/or cell type-dependent responses illustrate a lack of understanding of the intracellular mechanisms involved. Moreover, the high sequence specificity of siRNAs reduces their potential to efficiently target newly emerging strains. There is therefore an important need for the development of new strategies that display broad-spectrum properties against new influenza strains that arise through antigenic shift or drift.

Ribozymes are RNA molecules that possess catalytic activity, and both artificial and naturally occurring ribozymes function efficiently in human cells [19]–[22]. The hepatitis delta virus ribozyme (HDV-Rz) is derived from a natural motif found in HDV. Based on sequence complementarity, this ribozyme can specifically recognize and subsequently catalyze the cleavage of target RNA molecules. Its full activity under physiological conditions and outstanding stability *in vivo* makes it particularly attractive for the development as a therapeutic tool [23], [24]. Molecular engineering has led to the improvement of the HDV-Rz catalytic motif through the addition of a specific on/off adapter (SOFA) module, which increases the accuracy of the cleavage while reducing the cleavage of non-specific targets [25]. The resulting SOFA-HDV-Rz has already been successfully used in human cells to target and discriminate between closely related mRNAs [26], [27].

To evaluate the potential of a SOFA-HDV-Rz-based strategy to control influenza, we initially identified conserved target sequences in all subtypes known to infect the human population for six out of eight influenza collinear mRNAs, and generated a total of 45 candidate SOFA-HDV-Rz. The step-by-step screening strategy, consisting of both *in vitro* and cellular cleavage assay and *in cellulo* replication survey, led to the identification of efficient SOFA-HDV-Rz that were further characterized for their antiviral activity in an influenza mouse model.

## Results

### Conserved SOFA-HDV-Rz targets are present in six influenza segments

**Figure 1:**
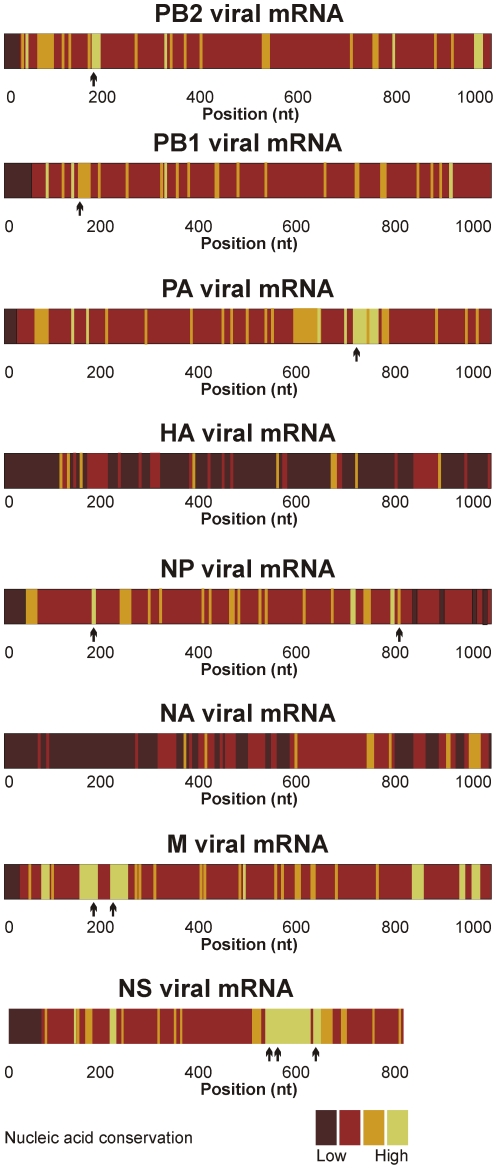
Representation of the conservation study performed on the viral mRNA fragments from human influenza A virus. For each of the 8 influenza mRNA fragments (PB2, PB1, PA, HA, NP, NA, M, NS), up to 20 nucleic acid sequences from the 7 human strains (H1N1, H1N2, H2N2, H3N2, H5N1, H7N3 and H9N2) were randomly selected and aligned to calculate a conservation rate at each position. The color gradation schematically identifies the level of conservation found in the first 1000 nucleotides of the different viral mRNAs. The arrows indicate the position of the most efficient ribozymes (see Table 1).

Even though the emergence of new influenza viruses is highly likely, the exact subtype and antigenic characteristics are currently impossible to predict. We therefore intended to identify regions in the viral mRNAs which are conserved in all subtypes that have the potential to cause disease in humans. Towards this, a bioinformatics analysis was conducted with complete genome sequences of 20 arbitrarily chosen isolates available in the NCBI Influenza Virus Resource database for each of the following subtypes: H1N1, H1N2, H2N2, H3N2, H5N1, H7N3, and H9N2. The sequences of each mRNA segment were aligned, and the degree of homology for the first 1000 nucleotides of each fragment was defined, since targeting the 5′ region of an mRNA is more likely to result in transcript fragments that can no longer encode a functional protein. Consistent with the high genetic variability of the viral glycoproteins [4], [28], neither the hemagglutinin (HA) nor the neuraminidase (NA) segments contained any conserved regions, while several conserved regions of sufficient size to be potential SOFA-HDV-Rz targets were identified for the other six segments (Figure 1).

**Figure 2:**
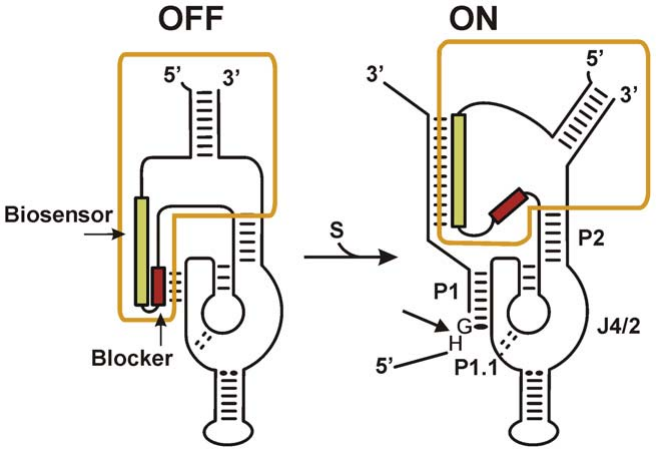
Schematic drawing of a SOFA-HDV-Rz. The yellow box indicates the SOFA module. The biosensor and the blocker are illustrated in green and red, respectively. The bold arrow indicates the cleavage site. S, P1, and P1.1 stand for the substrate, P1 recognition stem, and the P1.1 pseudoknot, respectively. Without the appropriate substrate, the cleaving activity of the SOFA-HDV-Rz is locked by the base pairing of the blocker (4 nts) on the P1 recognition sequence (OFF state). Upon recognition of its specific target, the biosensor (10–12 nts) of the SOFA module base pairs on the substrate downstream of the cleavage site, which disrupts the interaction of the blocker on the P1 recognition sequence and therefore allows the formation of the P1 stem (7 nts) between the ribozyme and the substrate (ON state). This leads to the specific cleavage of the RNA substrate.

SOFA-HDV-Rz are designed to contain three specific domains for a selected target: a blocker domain, which locks the catalytic core in an inactive conformation; a biosensor, which removes the block upon recognition of the target; and a P1 domain which binding results in cleavage of the targeted RNA (Figure 2). The SOFA module acts through the binding of the blocker on the P1 domain [25]: this short duplex increases the energetic barrier for non-specific interactions with substrates. The biosensor offers an extended base-pairing favouring interaction with the genuine substrate; this longer duplex disrupts the hindrance carried out by the blocker and therefore allows the P1 accessibility for elongated substrate binding and subsequent target cleavage. Screening of the conserved regions for the appropriate target sequences (HG*N*6-N3-6-N*9-12: H = A, C or T; N = A, T, G or C; * = conserved nt) yielded more than 45 potential SOFA-HDV-Rz. These candidates were then analyzed for possible off-targeting of cellular mRNAs using the RiboSubstrate software [29]. Only SOFA-HDV-Rz displaying a high level of specificity towards influenza mRNAs with a low probability of interactions with cellular mRNAs were retained for further investigation. The predicted specificity for each SOFA-HDV-Rz is shown in Table S1.

### A subset of SOFA-HDV-Rz shows catalytic activity

**Figure 3:**
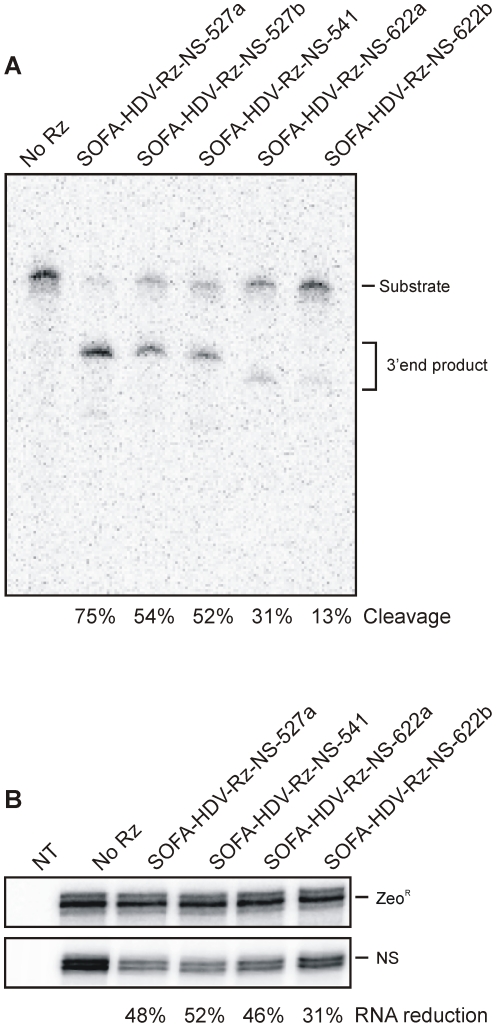
Catalytic activity of SOFA-HDV-Rz directed against influenza mRNAs. The identity of the SOFA-HDV-Rz tested, which contains the target name followed by a number indicating the cleavage site and a lower case when more than 1 ribozyme targets the same site, is listed above the gels. A negative control (No Rz) was performed in the absence of any SOFA-HDV-Rz. (A) 3′-radiolabeled RNA substrate consisting of the influenza NS mRNA was incubated with a SOFA-HDV-Rz at 37°C for 2 h in presence of magnesium in Tris-HCl buffer pH 7.5. The reaction products were separated on PAGE and visualized by phosphorimaging. The positions of the substrate and 3′ end product are indicated. (**B**) Ribonucleic protection assay on total cell extract prepared from Tet-modified HEK-293 cells transfected with pDUAL-JU expressing both the NS target (induced for 20 h) and the SOFA-HDV-Rz. The bands corresponding to the substrate mRNA are normalized with Zeo^R^ reference mRNA from the pDUAL-JU vector. The levels of Zeo^R^ and NS mRNAs are presented in the upper and lower panel, respectively.

To assess the ability of the selected SOFA-HDV-Rz to target specific influenza mRNA transcripts, the cleavage efficacy of radiolabeled *in vitro* synthesized H1N1 A/USSR/90/77 mRNAs was tested under single-turnover conditions ([Rz]>[S]) and analyzed on a denaturant polyacrylamide gel. Two active ribozymes were found to cleave the 5′ regions of both PB2 and PB1 mRNAs. The mRNA of PA was targeted by 11 SOFA-HDV-Rz while three different ribozymes were able to cleave both the nucleoprotein (NP) and matrix (M) mRNAs. Finally, all but one of the seven ribozymes selected against the NS mRNAs displayed catalytic activity (Figure 3A and Table 1). The SOFA-HDV-Rz-NS-527a was the most catalytically efficient molecule, displaying 75% cleavage of the viral NS RNA substrate. Overall, this assay yielded a collection of 26 SOFA-HDV-Rz that efficiently cleave the collinear mRNAs produced from the six remaining influenza segments.

**Table 1:** *In vitro* catalytic activity of the selected SOFA-HDV-Rz.

Target	SOFA-HDV-Rz	P1	BS	Cleavage %*
PB2	PB2-177	AUCAUCU	GGAUAUUUCAU	20
	PB2-324a	UUCCACU	GGUCCAUU	15
	PB2-324b	UUCCACU	GGUCCAUUUC	7
PB1	PB1-132a	CCAUGGU	GUAUCCUGUUCC	<1
	PB1-132b	CCAUGGU	AUCCUGUUCC	<1
	PB1-167a	UGUUGAU	UAUUGGUGUG	25
	PB1-167b	UGUUGAU	UGAAUAUUGGUG	<1
	PB1-296	CACAAUU	GCCAUUGCUUC	57
	PB1-907	GGUCAUU	GUGUCCUGAG	<1
PA	PA-134a	AUAUUGU	UCCAAGUGAG	38
	PA-134b	AUAUUGU	ACCUCCAAGUG	41
	PA-612a	UCGCCUU	CUUCAAUUGU	28
	PA-612b	UCGCCUU	UUUCUUCAAUUG	33
	PA-629	UUUCUUU	CCUGUGAUUUCA	49
	PA-714	UAGGCUU	CGAAUCCAUC	51
	PA-716	CAUAGGU	UUCGAAUCCA	54
	PA-722a	CAUCCAU	CCGUUCGGUU	42
	PA-722b	CAUCCAU	CCGUUCGGUUCG	43
	PA-724a	UCCAUCU	CGUUCGGUU	38
	PA-724b	UCCAUCU	AGCCGUUCGG	22
	PA-752a	UGCCCUU	GACAUUUGAGAA	9
	PA-752b	UGCCCUU	ACAUUUGAGA	11
	PA-754	CUUGCCU	UGGACAUUUG	<1
	PA-876a	AUCCAUU	AUGCUUAAUUUU	<1
	PA-876b	AUCCAUU	AAUGCUUAAUU	<1
NP	NP-59	UGGUGCU	GUUCAUAAGAC	<1
	NP-173a	GGUGCAU	CACUGAGUUU	52
	NP-173b	GGUGCAU	CUGAGUUUAAG	50
	NP-710a	GUUGCAU	UUUCCUUUGA	<1
	NP-710b	GUUGCAU	AAUUUUCCUUU	<1
	NP-744	UUUGUGU	UGAUCCAUCAUU	<1
	NP-804	GAUCUUU	AGUGCCAGAAA	54
M	M-172a	UCUUGUU	GAGGUGACAG	23
	M-172b	UCUUGUU	GAGGUGACAGGA	16
	M-226a	ACUGGGU	ACGCUGCAGUCC	7
	M-226b	ACUGGGU	GCAGUCCUCG	24
	M-231a	CGCUCAU	CUACGCUGCAG	<1
	M-231b	CGCUCAU	CGUCUACGCUGC	<1
NS	NS-110	GGGCAUU	AGCCGAUCAAG	<1
	NS-128	CGAAGCU	GGGACUUGAUC	34
	NS-527a	UAUGUCU	GACAUCCUCA	75
	NS-527b	UAUGUCU	UUUGACAUCC	54
	NS-541	GACAUCU	CCAAUUGCAU	52
	NS-622a	GAAUCUU	CUGCUUCUCC	31
	NS-622b	GAAUCUU	ACUGCUUCUC	13

*Margin of error on the cleavage rate is 10%.

### Several candidates SOFA-HDV-Rz retain activity in a cellular environment

To confirm the catalytic activity of the selected SOFA-HDV-Rz in a cellular environment, plasmids were designed to simultaneously express a SOFA-HDV-Rz and the corresponding target mRNA. SOFA-HDV-Rz were under the control of a cellular RNA polymerase III promoter, while viral mRNAs were expressed using a Tet-inducible Pol II promoter (Figure S1). We generated a HEK-293 cell line stably expressing the Tet-responsive transactivator, which supports activation of that promoter by doxycycline. That modified cell line was transfected with the dual vector expressing both the viral RNA and the corresponding SOFA-HDV-Rz.

Target mRNA expression was first detected after 4 h and peaked around 20 h after doxycycline induction (data not shown). To assess cleavage efficacy, total cellular RNA was extracted and analyzed by ribonucleic protection assay (RPA) using specific probes for each target mRNA as well as GAPDH and zeoR mRNA for cellular RNA and plasmid controls, respectively. Results were confirmed by Northern blot hybridization. The reduction in target mRNA levels remained constant between 4 h and 20 h of induction, *i.e.* at low and high target abundance. Of all the SOFA-HDV-Rz that showed activity for their respective synthetic target RNA, only one candidate retained activity in the cellular context for PB1, PB2, and the NP, while two candidates led to an appreciable reduction in the levels PA and M mRNAs, and four of the NS-specific SOFA-HDV-Rz remained active (Figure 3B). It should be noted that one NS-specific ribozyme (SOFA-HDV-Rz-NS-527b) could not be assayed since its primary sequence encompasses a Pol III stop signal. For all the SOFA-HDV-Rz that exhibited a significant cleavage activity, a P1.1 inactive mutant, which consists in replacing the nucleotides forming the pseudoknot I.I (i.e. including two GC bp) by for 4 adenosines, was constructed. These substitutions prevent the formation of the pseudoknot I.I, a crucial step in the folding pathway, thereby inhibiting its catalytic activity [30], [31]. Using such controls, we observed in most of the cases a reduction in the overall levels of the targeted mRNAs in cellular assays (data not shown) that most likely results from antisense effect, since those mutants retain the ability to fully bind the target. For example, the active and inactive SOFA-HDV-Rz-NP-804 displayed a 38% and 24% reduction of the target mRNA, respectively. More importantly, the reductions observed with the active ribozymes were more significant than their respective inactive version in all cases (data not shown), supporting that they resulted from the cleavage action.

### Combined SOFA-HDV-Rz displays antiviral activity

**Figure 4:**
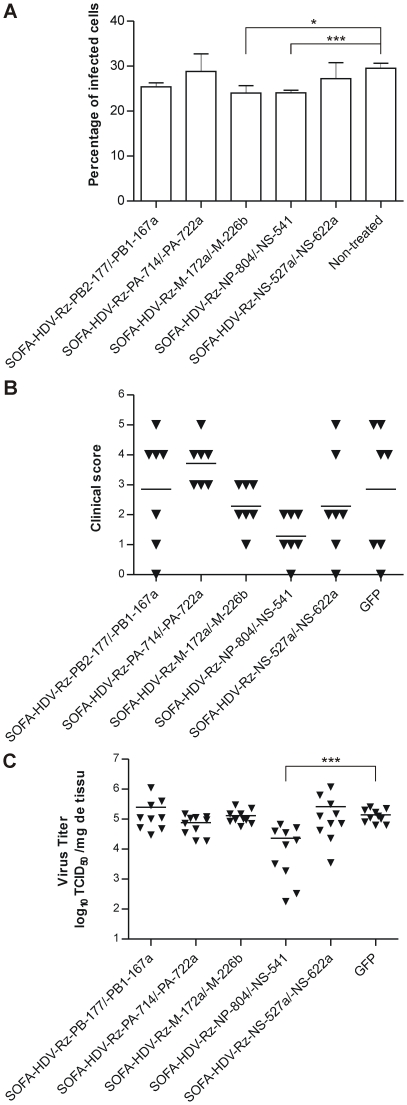
Antiviral activity of SOFA-HDV-Rz (A) in cells and (B and C) *in vivo*. (**A**) MDCK cells were transfected with the indicated SOFA-HDV-RZ combinations, and infected with PR8 at a MOI of 0.01 after 24 h. Ten hours later, the cells were fixed and stained with a monoclonal antibody against HA. The percentage of HA-positive cells detected in three independent experiments is shown. Error bars indicate standard deviation. (**B** and **C**) BALB/c mice were transfected intranasally with the indicated SOFA-HDV-Rz combinations and challenged with 10^5^ 50% tissue culture infectious doses (TCID_50_) of PR8 6 h later. (**B**) Clinical score on day 3 after infection. The clinical score represents a combination of posture, activity, and weight loss scores evaluated on a 0-1-2 scale as detailed in materials and methods. Results from 2 independent experiments are shown, and each symbol represents one animal. (**C**) Viral lung titers on day 1 after infection. Animals were sacrificed 24 h after infection and the lung was harvested. Titers are expressed as TCID_50_ per g of tissue. Student's t-test : asterisk, P<0.05; three asterisks, P<0.001.

To evaluate the antiviral activity of the catalytically active SOFA-HDV-Rz, we established a flow cytometry-based assay allowing us to quantify the proportion of infected cells. Toward this, Madin-Darby canine kidney (MDCK) cells, which are naturally susceptible to influenza infection, were first transfected with SOFA-HDV-Rz encoding plasmids and then infected with H1N1 A/Puerto Rico/8/34 (PR8) at a multiplicity of infection (MOI) of 0.1. An antibody against the HA protein was chosen to ensure an unbiased detection of infected cells, since none of the ribozymes target this particular glycoprotein. Moreover, combinations of two SOFA-HDV-Rz targeting the same or different viral mRNAs were transfected simultaneously. Ten hours after infection, around 30% of the non-treated cells expressed the HA protein (Figure 4A). While no improvement was seen for the combination of PA- and NS-targeting ribozymes, both the SOFA-HDV-Rz-M-172a/-M-226b and -NS-541/-NP-804 pairs resulted in a small reduction in the number of HA-positive cells (Figure 4A). No significant effect was observed for individually transfected ribozymes (data not shown).

### Only the NS/NP combination retains antiviral activity *in vivo*

The previous result does not provide any information on the actual number of viral particles produced in the presence of active ribozymes. It only indicates that a smaller number of infected-cells are actually detected in the presence of certain ribozyme combinations. Considering that a partial reduction of viral titer can potentially cause a significant decrease in disease severity since the immune system contributes importantly to the control of influenza replication and spread *in vivo*, we further investigated the extent of morbidity reduction and viral inhibition by the SOFA-HDV-Rz combinations in a mouse model. To validate the *in vivo* transfection protocol, mice were initially transfected intranasally with an eGFP expression plasmid using In Vivo-JetPEI as transfection agent. Twenty-four hours later, eGFP expression was detected throughout the lower respiratory tract, confirming efficient delivery to the site of influenza infection (data not shown). To assess *in vivo* efficacy, the different SOFA-HDV-Rz combinations were instilled intranasally, followed by infection with 10^5^ 50% tissue culture infectious doses (TCID_50_) of PR8 6 h later. Considerable variability of clinical disease severity was observed in groups treated with the control eGFP plasmid or the SOFA-HDV-Rz-PA-714/-PA-722a, -PB1-167a/-PB2-177, and -NS-527a/NS-622a combinations, while all animals in the –M-172a/-M-226b and -NS-541/-NP-804 treated groups were protected from severe disease and death (Figure 4B). Mice transfected with the SOFA-HDV-Rz-NS-541/-NP-804 combination displayed especially lower clinical scores than the average controls. To examine the direct inhibitory effect on virus replication, animals were transfected with the same SOFA-HDV-Rz combinations, followed by infection with PR8 after 6 h and sacrifice 24 h after infection. Quantification of viral titers in lung tissue revealed that only the SOFA-HDV-Rz-NS-541/-NP-804 combination significantly inhibited virus replication at early times after infection, with up to 1000-fold reduction in viral titer seen in some individuals (Figure 4C).

## Discussion

We have developed a step-by-step strategy to achieve efficient and affordable selection of therapeutic molecules against conserved regions of a broad range of influenza A subtypes. This strategy capitalizes on an *in silico* analysis of all subtypes known to infect the human population, allowing an inexpensive and time-saving identification of regions that are conserved over time and regardless of reassortments. We designed SOFA-HDV-Rz to target those identified regions and catalytically cleave the mRNA, which are subsequently degraded by the host cellular machinery. The most promising candidates were further evaluated first in *in vitro* assays to assess catalytic activity, and then regarding their antiviral activity in cell culture and *in vivo*, yielding two combinations with promising prophylactic activity. Our study thus provides proof-of-principle for the identification of candidate HDV-SOFA-Rz with antiviral activity against a highly variable RNA virus.

The lack of proof-reading capacity of the viral polymerase, the high replication rate and short replication time are the cause of the high genetic variability inherent to RNA viruses, unless there is functional pressure to maintain specific nucleotides [28], [32]. The extensive *in silico* conservation study, which to our knowledge is the first of its kind, allowed the identification of several such stretches of conserved nucleotides sufficiently long for efficient SOFA-HDV-Rz targeting in all but the two viral mRNAs coding for the viral glycoproteins NA and HA. These two major surface glycoproteins are highly variable due to the continuous antigenic pressure [4], [28], [33] and were thus excluded from the study. Therapeutic molecules targeting regions identified through this process are thus expected to display broad-spectrum specificity and are likely to retain efficacy against new strains, thereby overcoming the main weakness of all antiviral strategies targeting nucleotide sequences [19], [34]–[36].

The HDV-Rz is one of the rare examples derived from an RNA species found to infect human cells, therefore being naturally optimized to fully function in physiological conditions [23], [24]. A SOFA module has been engineered to improve its catalytic characteristics, greatly increasing its fidelity [25]. The *in vitro* activity of many SOFA-HDV-Rz designed to target conserved regions identified with the bio-informatics of six collinear viral mRNA was evaluated, as targeting the viral mRNA might be more effective than the genomic RNA due to its higher abundance and lack of encapsidation by the NP protein [36]. Since RNA molecules can have long-range interactions, working with *in vitro* synthesized full-length target RNA is likely to be an accurate way to evaluate the potential of the effective candidates in regards of the tridimensional accessibility. Roughly half of the potential candidates showed appreciable catalytic activity, which is in the same range as other studies done with long RNA and SOFA-HDV-Rz [27], [37], [38]. The ineffectiveness of the rejected candidates could be explained either by inaccessibility of the target RNA or by kinetic trapping of the SOFA-HDV-Rz in an inactive conformation, which could be attributed to diverse interaction between different parts of the molecule. In particular, the biosensor interaction with the J4/2-P2 region (Figure 2) is known to inhibit the catalytic activity. Such example is found in the SOFA-HDV-Rz-NP-710a and its 7 consecutive nts that could be base-paired with that area. An elevated blocker strength is also detrimental for the release of the P1 and successive substrate recognition. This situation is found in SOFA-HDV-Rz-PB1-132a and -PB1-132b that have an elongated blocker (from the continuous biosensor nts) of 6 nts containing 4 strong G-C bonds. With the high potential of this novel class of therapeutic ribozyme, it would be of great interest to learn which criteria could discriminate between an intrinsically functional or inactive SOFA-HDV-Rz. The information gathered from the SOFA-HDV-Rz panel produced in this project is currently under investigation to develop new screening and selection criteria to improve the yield of active molecules in future studies.

Around half of the catalytically active SOFA-HDV-Rz failed to display sufficient activity in cellular assays, either due to a differential reaching of the target (divergent conformation or protection through interaction with host factors) or to an inhibited state of the ribozyme. The observed effect of the active candidates is necessarily the sum of antisense and cleavage action, leading to the development of powerful antisense-enhanced ribozymes [19], [39]. However, the antisense effect in some cases was really minimal while all ribozymes were shown to actively cleave the mRNAs, although at different levels. The use of certain combinations of ribozymes clearly reduces the probability of generating escape mutants [35], [40]. Therefore, combinations of SOFA-HDV-Rz chosen from the collection of catalytically active molecules were tested for their antiviral effect in infected cells. While most of the combinations targeting the same RNA showed no increase in activity, all the SOFA-HDV-Rz duos targeting different viral mRNAs inhibited viral growth, indicating a synergic effect. However, it is too early in our investigation to comment on how the synergic effect precisely takes place and/or on the steps of the viral replication cycle that are the most affected by the action of the ribozyme. The slight decrease of infected cells in the replication assays is explained by the sorting system which scores positive for an infected cell independently of its viral titer (Figure 4A). The efficient SOFA-HDV-Rz combinations also reduced viral replication in lungs and protected animals from severe illness, whereas combinations that were unable to inhibit viral replication in cell culture had no beneficial effect *in vivo*. The SOFA-HDV-Rz NS-541/NP-804 duo led to a one order magnitude difference in peak lung titers, which is in a range previously shown to correlate with decreased disease severity [36]. It will be of great interest to investigate further combinations and to extend the clinical evaluation to post-exposure treatment in order to gain insight in the curative prospect of SOFA-HDV-Rz. Respiratory viruses such as influenza A are ideal target for nucleic acid therapy since the upper airways and even lungs are relatively accessible for gene delivery systems [36].

In summary, our study constitutes a proof-of-principle for a new strategy to find therapeutic molecules against highly variable RNA viruses using influenza A as a prototype. Our approach offers an inexpensive and time-saving way for the development of any RNA recognition-based design, starting with the identification of conserved nucleic acid regions by *in silico* analysis. A step-by-step selection strategy with increasing stringency from *in vitro* catalytic cleavage evaluation to *in vivo* efficacy assessment yielded a panel of SOFA-HDV-Rz that target influenza A virus mRNAs. This innovative system led to the identification of potent SOFA-HDV-Rz against which emergence of resistance would be unlikely giving the conserved nature of the targeted nucleotides.

## Materials and Methods

### Bioinformatics analysis

For each of the eight viral mRNA fragments (PB2, PB1, PA, HA, NP, NA, M, NS), 20 full-length sequences from the NCBI Influenza Virus Resource [41] were chosen arbitrarily for each of the seven subtypes known to infect humans (H1N1, H1N2, H2N2, H3N2, H5N1, H7N3, H9N2) resulting in a total of about 2000 sequences analyzed. Sequences were aligned and analyzed for conserved sequences fulfilling the requirement for a SOFA-HDV-Rz target (HG*N*_6_-N_3–6_-N*_9–12_; H = A, C or T; N = A, T, G or C; * = conserved nt). The sequence of the each candidate SOFA-HDV-Rz designed was screened for the potential off-targeting on human host mRNAs using the RiboSubstrate software [29] with the Human NCBI build36.2 mRNAs database (ftp://ftp.ncbi.nih.gov/genomes/H_sapiens/RNA) and standard settings (BS mismatch = 3; Minimum spacer length = 1; Maximum spacer length = 10).

### RNA synthesis

DNA oligonucleotides (IDT) with the following sequences were used for the synthesis of SOFA-HDV-Rz: a specific SOFA-HDV-Rz_fwd 5′-TAATACGACTCACTATAGGGCCAGCTAGTTT-BS_9–12_-BL_4_-CAGGGTCCACCTCCTCGCGGT-P1_7_-GGGCATCCGTTCGCG-3′ (where BS, BL and P1 indicate the biosensor, the blocker sequence and the P1 recognition domain, respectively; T7 promoter is in the underlined region) and the universal SOFA-HDV-Rz_rev 5′-CCAGCTAGAAAGGGTCCCTTAGCCATCCGCGAACGGATGCCC -3′. The double-stranded SOFA-HDV-Rz DNA was obtained after a filling reaction, and SOFA-HDV Rz RNA was synthesized by a run-off transcription from PCR products as described previously [25]. Briefly, transcriptions were performed in the presence of purified T7 RNA polymerase (10 µg) [42] as described previously [43], RNaseOUT (20 U, Invitrogen), pyrophosphatase (0.01 U, Roche Diagnostics, Laval, QC, Canada) and PCR product (2–5 mM) in a buffer containing 80 mM HEPES-KOH pH 7.5, 24 mM MgCl_2_, 2 mM spermidine, 40 mM DTT, 5 mM of each NTP in a final volume of 100 ml at 37°C for 2 h. Upon completion, the reaction mixtures were treated with DNase RQ1 (Amersham Biosciences, Piscataway, NJ) at 37°C for 20 min, and the RNA was purified by a phenol∶chloroform extraction and then precipitated with ethanol.

Influenza cDNAs of H1N1 A/USSR/90/77 were subcloned in pBluescript SK+ (Stratagene). RNA substrates were synthesized by run-off transcription from SacII (NEB) linearized pBluescript-SK+ containing the appropriate cDNA template with the MEGAscript T7 transcription kit (Ambion) according to the manufacturer instructions. For the production of internally radiolabelled substrate, the transcription reactions were performed in the presence of 5 uM UTP and 6 pmol of α-^32^P UTP (3000 Ci/mmol, Perkin Elmer). Upon completion, the reaction mixtures were treated with TURBO DNase (2 U, Ambion) at 37°C for 20 min. The RNA products were fractionated by denaturing polyacrylamide gel electrophoresis (PAGE; 19 ∶ 1 ratio of acrylamide to bisacrylamide). The reaction products were visualized by either UV shadowing or autoradiography. The bands corresponding to the correct size were cut out and the transcripts eluted overnight at 4°C. The 3′ radiolabelled substrates were generated in a ligation reaction performed with 50 pmol of purified RNA, 30 pmol of ^32^PcP (3000 Ci/mmol, Perkin-Elmer), 20 U of RNaseOUT (Invitrogen) and purified T4 RNA ligase (10 µg) in a buffer containing 50 mM Tris-HCl pH 7.8, 10 mM MgCl_2_, 1 mM ATP, 10 mM DTT and 10% DMSO in a final volume of 10 µL at 37°C for 1 h. The products were fractionated by denaturing PAGE, isolated and purified as described above.

### *In vitro* cleavage assays

SOFA-HDV-Rz cleavage assays were performed in the presence of 1 pmol of either 3′- (NS substrate) or internally ^32^P-labelled target mRNA (all other substrates) mixed with SOFA-HDV-Rz (20 pmol) in a 20 µL mixture containing 50 mM Tris-HCl, pH 7.5, and 10 mM MgCl_2_ and then incubated at 37°C for 2 h. The reactions were stopped by the addition of denaturing loading buffer, separated on denaturing 5% PAGE gels, and then analyzed by autoradiography on a Phosphorscreen (GE Healthcare).

### Construction of pDUAL-JU and introduction of SOFA-HDV-Rz and target Influenza mRNAs

The tet-responsive Pol II promoter Ptight was amplified from the plasmid pTRE-Tight (Clontech Laboratories, Inc.), cloned in pGEM-T (Promega) and subcloned in pBudCE4.1 (Invitrogen) with the added restriction sites BbsI and NheI (NEB). The Pol III promoter PhHI was amplified from the plasmid psiRNA-hH1GFPzeo (InvivoGen) and cloned in the engineered plasmid with the added restriction sites BspHI and SbfI. The two promoters inserted in the resulting pDUAL-JU vector were sequenced in both directions to assure that the correct sequences were cloned. The different SOFA-HDV-Rz were PCR amplified and inserted after the PhHI promoter of pDUAL-JU using added BbsI restriction sites. The PCR products containing the respective targets were cloned with added MluI and NotI restriction sites after the Ptight promoter of the pDUAL-JU containing or not the DNA sequence of a SOFA-HDV-Rz. The resulting plasmids were sequenced to verify both the sequences of the SOFA-HDV-Rz and the substrate.

### Cell culture and DNA transfection

HEK-293 cells (obtained from the American Type Culture Collection; CRL-1573) were grown in Dulbecco's modified Eagle's medium (DMEM, Wisent) supplemented with 10% fetal bovine serum (FBS, Wisent), 1 mM sodium pyruvate and 2 mM L-glutamine at 37°C in 5% CO2. Cells were transfected with pTET-ON (Clontech) using Lipofectamine 2000 (Invitrogen), as per the manufacturer's instructions. This pTET-ON vector is used to transform cells so they can express the Tet-responsive transactivator. Briefly, cells were seeded into 10 cm-dishes plates at 3×106 cells/well and transfected with 24 µg of plasmid DNA that was complexed with the provided transfection reagent. They were then selected for resistance to G-418 at a final concentration of 600 µg/mL. G-418-resistant colonies were isolated and screened for the highest fold-induction (highest expression with lowest background) with the luciferase reporter assay kit provided by the manufacturer (Clontech).

HEK-293 cells expressing the Tet-responsive element were transfected with the different pDUAL-JU constructs containing the target with or without SOFA-HDV-Rz. Briefly, cells were seeded into 12-well plates at 2×10^5^ cells/well and transfected with 1 µg of plasmid DNA that was complexed with Lipofectamine 2000. The target mRNA expression was induced for 4 or 20 h with doxycycline (Sigma) at a final concentration of 975 ng/mL prior to RNA extraction with TriPur (Roche) according to the manufacturer instructions.

### Ribonucleic Protection Assay and Northern Blot Hybridizations

For the ribonucleic protection assay (RPA), 5 µg of total RNA extract was processed with the RPA III™ Ribonuclease Protection Assay Kit (Ambion) as recommended by the manufacturer. Probes with 3′ and 5′ overhangs of 15 nucleotides were transcribed from PCR products of pBluescript plasmids containing the fragments described above.

Northern blot analyses of 10 µg total RNA extract were performed as described previously [25]. The probes to detect the Influenza target or the control Zeo^R^ mRNA were obtained by a T3 RNA polymerase-based transcription in the presence of 30 pmol of α-^32^P UTP (3000 Ci/mmol, Amersham Biosciences) on the pBluescript plasmids containing the fragments described above, and the β-actin RNA probe was transcribed from pTRI-B-Actin-Mouse (Ambion). The plasmids were digested to yield run-off transcription products ranging from 134 nts to 503 nts in length. All probes were prepared with the MAXIscript T3 transcription kit (Ambion) according to the manufacturer instructions, and hybridizations were carried out for 16–18 h at 60°C. The membranes were then washed and analyzed by autoradiography on a Phosphorscreen (GE Healthcare).

### Evaluation of antiviral activity in cellulo

Madin-Darby canine kidney (MDCK) cells (obtained from the American Type Culture Collection; CCL-34) were seeded in 12-well plates at 4×10^5^ cellules/ml in DMEM supplemented with 5% FBS. The next day, the cells were transfected with 0.75 µg of two pDUAL-JU plasmids coding for different ribozymes, but free of any substrate sequence, using Lipofectamine 2000. On day 2, the medium was replaced by DMEM with 2 µg/ml TPCK-trypsin (Sigma), and the cells were infected with H1N1 influenza A/Puerto Rico/8/34 (A/PR/8/34) at a multiplicity of infection (MOI) of 0.1. After 10 h, cells were detached using Versene (Invitrogen), washed once with phosphate-buffered saline (PBS, Invitrogen), and fixed with Cytofix/Cytoperm (BD Biosciences). After incubation with a mouse monoclonal antibody against influenza hemagglutinin (clone C102; Santa Cruz Biotech) in combination with a goat anti-mouse Alexa647-labeled secondary antibody (Invitrogen), the percentage of infected cells was quantified by flow cytometry.

### *In vivo* efficacy assessment

Groups of three to five 6 week-old female BALB/c mice (Charles River) were instilled intranasally with 10 µg each of the same plasmid combinations used for the *in vitro* replication experiments, or 20 µg of pEGFP-N1 (Clontech) as transfection control, using In Vivo-jetPEI™ (Polyplus Transfection) as transfection reagent according to the manufacturer's instructions, followed intranasal infection with 10^5^ TCID_50_ of H1N1 A/PR/8/34 6 h later. Animals were monitored daily for signs of disease. To assess clinical disease severity, a three-step grading system was used.

For Posture, 0 represented normal, 1 slow movement, and 2 back curved and tousled fur. Activity was graded 0 for normal, 1 for calm, and 2 for inactive, and weight loss was classified as 0 for 0 to 15% loss, 1 for 15 to 20% loss, or 2 for more than 20%.

In a second experiment, all animals were euthanized on day 1 after infection, the lungs were harvested, and virus titers were determined by limited dilution method and expressed as TCID_50_. The Mann-Whitney test was used for statistical analyses. All experiments were approved by the institutional animal care and use committee of the Institut National de la Recherche Scientifique-Institut Armand-Frappier (#0705-04).

## Supporting Information

Figure S1 **Schematic representation of the plasmid designed to simultaneously express a SOFA-HDV-Rz and the corresponding target mRNA.** SOFA-HDV-Rz are under the control of a cellular RNA polymerase III promoter, while viral mRNAs are expressed using a Tet-inducible Pol II promoter.
(TIF)

Table S1 **Predicted specificity of the selected SOFA-HDV-Rz.**
(EPS)
