# Supplementary figures and images for: A Novel Ribozyme-Based Prophylaxis Inhibits Influenza A Virus Replication and Protects from Severe Disease
Source: PLoS One. 2011 Nov 14;6(11):e27327. doi: 10.1371/journal.pone.0027327 (PMC3215696; doi:10.1371/journal.pone.0027327)

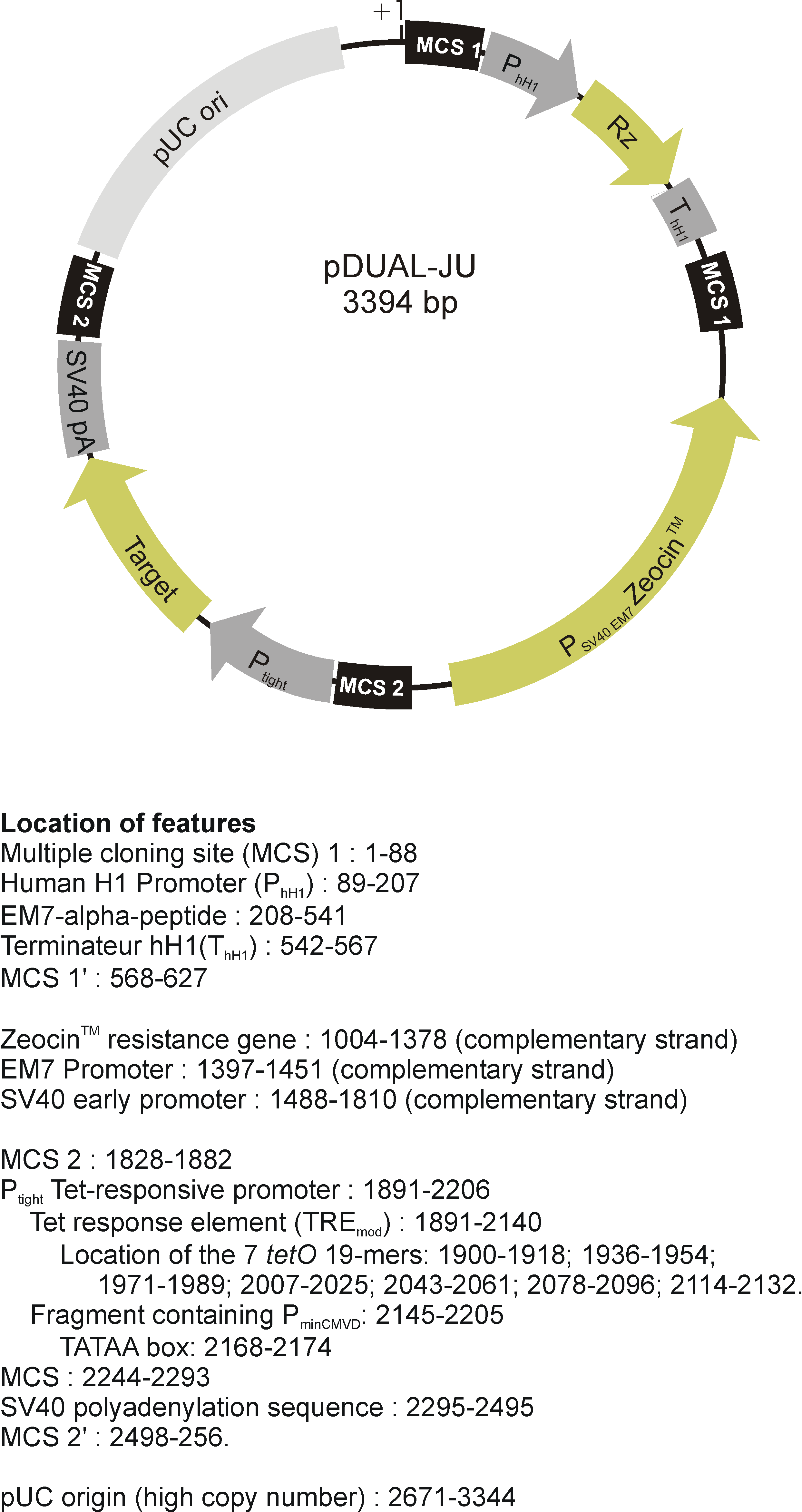

Supplement: Figure S1 — Schematic representation of the plasmid designed to simultaneously express a SOFA-HDV-Rz and the corresponding target mRNA. SOFA-HDV-Rz are under the control of a cellular RNA polymerase III promoter, while viral mRNAs are expressed using a Tet-inducible Pol II promoter. (TIF) [file pone.0027327.s001.tif]
